# Influencing and moderating factors analyzed in the group art therapy of two schizophrenic inpatients

**DOI:** 10.7603/s40681-015-0024-7

**Published:** 2015-11-28

**Authors:** Chung-Chieh Hung, Yung-Wen Ku

**Affiliations:** 1 Graduate Institute of Clinical Medical Science, College of Medicine, China Medical University, No. 91, Hsueh-Shih Road,, 404 Taichung, Taiwan; 2 Department of Psychiatry, China Medical University Hospital, 404 Taichung, Taiwan; 3 Department of Psychiatry, Changhua Christian Hospital, 500 Changhua, Taiwan

**Keywords:** Art therapy, Schizophrenia, MATISSE study

## Abstract

Art therapy has been considered a guideline treatment for schizophrenia. Due to difficulty in the outcome measurement, the research is difficult and controversial. Here, we presented two schizophrenic patients receiving the regular art group therapy. We compared their characteristics and different outcome. Art therapy is difficult to quantify. However, we could qualify the improvement from the individual case. Further study might be focus on how to make appropriate qualification of art therapy and individualized difference instead of enrollment of huge data bank.

## 1. Introduction

Although art therapy has been considered a guideline treatment for schizophrenia [1, 2], the reviewed article shows an inconsistent result [3] .

Here, we have presented two schizophrenic patients that received semi-structured art therapy by one trained psychiatrist. We have analyzed their clinical symptoms, psychosocial problems, and the images during the therapy.

All images were made with informed consent for publication and in anonymity.

## 2. Case reports

A 19-year-old, patient A was diagnosed with schizophrenia 4 years ago, with the presentation the disease being auditory hallucinations with voices commenting, referential delusions, and delusion of thought. She originally studied in the special education department of a university but stopped due to mental illness. Due to the aggravation of her psychotic symptoms and violent attempts toward her family, she was admitted to our acute psychiatric ward. Under a daily treatment of 15 mg of aripiprazole, she became relatively stable and began to take part in a semistructured art therapy group weekly. She initially drew a girl with wonder in a white skirt on a green lawn. With the progression of time, her drawings became more abundant and integrated. She also began to become involved in the story telling of her creations. The themes started from the imprisoned long-hair princess in the high tower of a castle and the collage work she did by herself. After receiving the pharmacologic and art therapy treatment for around one month, the patient was relatively stable and was able to reestablish a good relationship with her family. Her Positive and Negative Syndrome Scale (PANSS) [4] was improved from 90 to 65 and Scale for the Assessment of Negative Symptoms (SANS) [5] went from 69 to 45.

Patient B is a 37-year-old housewife suffering from schizophrenia for the past 10 years with the manifestations of the disease being disorganized behavior, disorganized speech, auditory hallucinations, and olfactory hallucinations. She was compulsorily admitted to our acute psychiatric ward according to the Mental Health Act of Taiwan due to aggravation of disturbing and violent behavior. She received a risperidone consta depot injection because she refused treatment. What’s interesting is that she did express interest in participating in the semi-structured art therapy group. She enjoyed using crayons to freely associate the theme denoted by the therapist. Her pictures were all of poorly understood symbols and religious subjects. Her PANSS improved from 114 to 92 and her SANS went from 94 to 69.

The semi-structured art therapy was performed by the trained psychiatrist, with a frequency of four times every month. Each session’s guiding sentence for what the patients should draw were similar and used the photo card collection that was published by the Taiwan Art Therapy Association (TATA). The therapist used cards full of images, and the patients drew their favorite images. After completion of their works, the therapist invited them to relate the themes of their pictures.

**Table 1:** A comparison of the two patients.

Variables	Patient A	Patient B
Age	19	37
Gender	Female	Female
Education	Senior high school	Junior high school
Occupation	Student	Housewife
Illness duration (years)	4	10
Schizophrenia subtype (DSM-IV)	paranoid	disorganized
PANSS improvement	90→65	114→92
SANS improvement	69→45	94→69
Medication	Oral atypical antipsychotics	Injection depot
Favorite theme(s)	Fairy tales	Religion, Symbols
Frequency of art therapy (times/month)	4	4
Key family person	Parents	Husband
Psychosocial stressor	Sibling conflicts	Marital problems

**Image 1:**
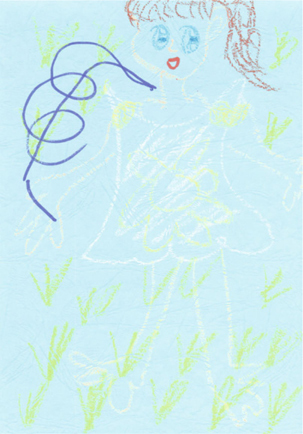
from the patient A, with the title of “The Dancer on the Earth”

**Image 2:**
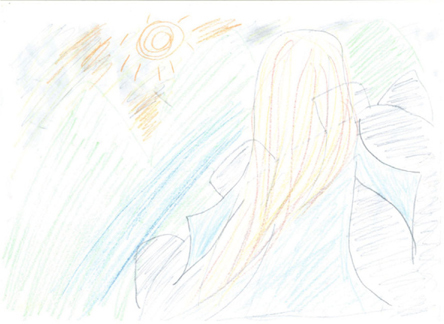
from the patient A with the title of “The Long-Haired Princess Imprisoned in the Castle”

**Image 3:**
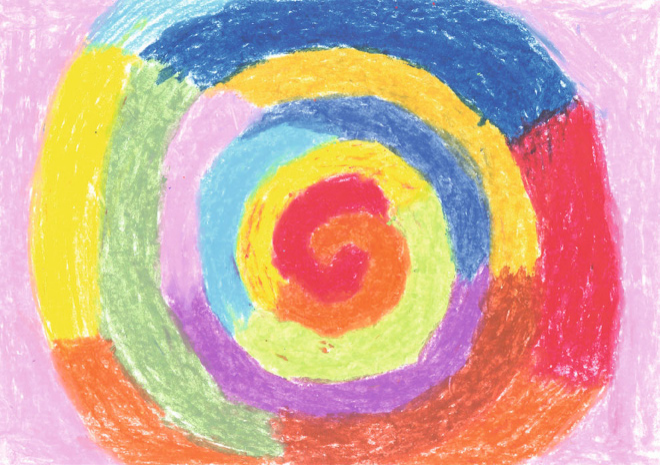
from the patient B with the tile of “Candy Time Machine”

## 3. Discussion

From the images created by patient A and B, individual differences among their age, education, occupation, clinical symptoms, and even schizophrenia subtypes as defined by the DSM-IV [6] can be discerned. In summary, we outlined the comparison and difference from these two patients in the Table 1.

The literature reviews are consistent with our results that the better outcome as measured from the PANSS in clinical symptoms seem to be associated with a patient’s younger age, higher level of functioning before the mental illness, paranoia type, and a lower SANS [7, 8]. In schizophrenic patients, improvement in negative symptoms predicted changes in Neuroticism and (inversely) in Extraversion and Openness [9].

Eligibility criteria in the previous MATISSE study were broad. For the small effect observed when group art therapy was offered to all patients with a diagnosis of schizophrenia, and the resources involved in delivering such an intervention, it is important to identify patients who appear the most likely to benefit from such therapy [10].

**Image 4:**
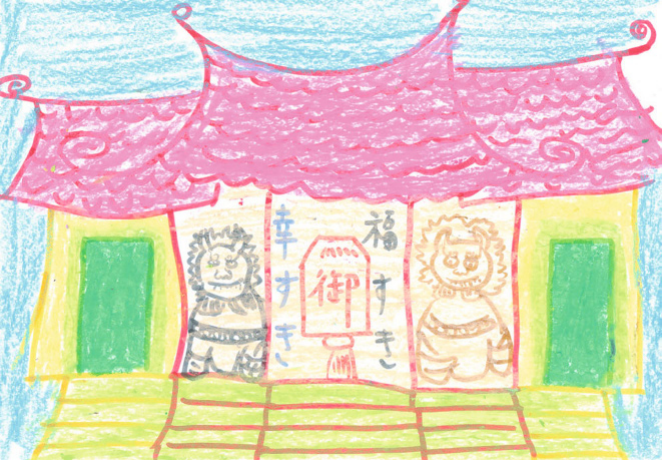
from from the patient B with the tile of “One Buddha Temple”

From the two patients presented here, we might see that motivation plays an important role in improving the clinical symptoms of schizophrenics. Another interesting and novel finding in these two patients was that the younger patient tended to be involved in story telling style in the group art therapy while the older patient appeared to be more involved in her own fantastic and strange world.

Due to both the variety of art therapy and patient subgroups, further study might be needed to fully elucidate the benefits of group art therapy in patients suffering from schizophrenia.
